# *TERT* Promoter Mutations and Their Impact on Gene Expression Profile in Papillary Thyroid Carcinoma

**DOI:** 10.3390/cancers12061597

**Published:** 2020-06-17

**Authors:** Dagmara Rusinek, Aleksandra Pfeifer, Marta Cieslicka, Malgorzata Kowalska, Agnieszka Pawlaczek, Jolanta Krajewska, Sylwia Szpak-Ulczok, Tomasz Tyszkiewicz, Monika Halczok, Agnieszka Czarniecka, Ewa Zembala-Nozynska, Mykola Chekan, Roman Lamch, Daria Handkiewicz-Junak, Aleksandra Ledwon, Ewa Paliczka-Cieslik, Aleksandra Kropinska, Barbara Jarzab, Malgorzata Oczko-Wojciechowska

**Affiliations:** 1 Department of Genetic and Molecular Diagnostics of Cancer, Maria Sklodowska-Curie National Research Institute of Oncology Gliwice Branch, 44-102 Gliwice, Poland; Aleksandra.Pfeifer@io.gliwice.pl (A.P.); Marta.Cieslicka@io.gliwice.pl (M.C.); Malgorzata.Kowalska@io.gliwice.pl (M.K.); Agnieszka.Pawlaczek@io.gliwice.pl (A.P.); Tomasz.Tyszkiewicz@io.gliwice.pl (T.T.); Monika.Halczok@io.gliwice.pl (M.H.); Malgorzata.Oczko-Wojciechowska@io.gliwice.pl (M.O.-W.); 2 Department of Nuclear Medicine and Endocrine Oncology, Maria Sklodowska-Curie National Research Institute of Oncology Gliwice Branch, 44-102 Gliwice, Poland; Jolanta.Krajewska@io.gliwice.pl (J.K.); Sylwia.Szpak-Ulczok@io.gliwice.pl (S.S.-U.); Daria.Handkiewicz-Junak@io.gliwice.pl (D.H.-J.); Aleksandra.Ledwon@io.gliwice.pl (A.L.); Ewa.Paliczka@io.gliwice.pl (E.P.-C.); Aleksandra.Kropinska@io.gliwice.pl (A.K.); Barbara.Jarzab@io.gliwice.pl (B.J.); 3 Department of Oncological and Reconstructive Surgery, Maria Sklodowska-Curie National Research Institute of Oncology Gliwice Branch, 44-102 Gliwice, Poland; Agnieszka.Czarniecka@io.gliwice.pl; 4 Tumor Pathology Department, Maria Sklodowska-Curie National Research Institute of Oncology Gliwice Branch, 44-102 Gliwice, Poland; Ewa.Zembala-Nozynska@io.gliwice.pl (E.Z.-N.); Mykola.Chekan@io.gliwice.pl (M.C.); Roman.Lamch@io.gliwice.pl (R.L.)

**Keywords:** *TERT*, papillary thyroid carcinoma, gene expression profile

## Abstract

Background: Telomerase reverse transcriptase promoter (*TERT*p) mutations are related to a worse prognosis in various malignancies, including papillary thyroid carcinoma (PTC). Since mechanisms responsible for the poorer outcome of TERTp(+) patients are still unknown, searching for molecular consequences of *TERT*p mutations in PTC was the aim of our study. Methods: The studied cohort consisted of 54 PTCs, among them 24 cases with distant metastases. *BRAF* V600E, *RAS,* and *TERT*p mutational status was evaluated in all cases. Differences in gene expression profile between TERTp(+) and TERTp(−) PTCs were examined using microarrays. The evaluation of signaling pathways and gene ontology was based on the Gene Set Enrichment Analysis. Results: Fifty-nine percent (32/54) of analyzed PTCs were positive for at least one mutation: 27 were BRAF(+), among them eight were TERTp(+), and 1 NRAS(+), whereas five other samples harbored *RAS* mutations. Expression of four genes significantly differed in BRAF(+)TERTp(+) and BRAF(+)TERTp(−) PTCs. Deregulation of pathways involved in key cell processes was observed. Conclusions: *TERT*p mutations are related to higher PTC aggressiveness. *CRABP2* gene was validated as associated with *TERT*p mutations. However, its potential use in diagnostics or risk stratification in PTC patients needs further studies.

## 1. Introduction

Papillary thyroid carcinoma (PTC) is the most common type of thyroid cancer (TC), generally characterized by a good prognosis. Only a small percentage of PTCs demonstrates a higher aggressiveness and poor outcomes [1]. A proper stratification is crucial to select a group of patients at high risk of unfavorable PTC course and simultaneously to avoid overtreatment in low-risk cases. Moreover, the intra-tumor and interpatient heterogeneity observed in PTC, seem to have important implications for therapeutic approaches [2,3]. Despite significant progress in our understanding of the molecular background of PTCs, reliable prognostic and predictive molecular markers are still lacking. The Cancer Genome Atlas (TCGA) study, published in 2014, substantially reduced the ‘dark matter’ of PTC genome up to 3.5%, as genetic alterations were defined in about 96.5% PTC cases [4]. The most common ones, as reported previously by other authors, were related to genes of the RAS/RAF/MAPK pathway, with the *BRAF* V600E mutation being present in 61.7% of PTCs, *RAS* mutations in 12.9%, and *RET/PTC* rearrangements in 6.8% of cases. The mutation density in the analyzed cohort was low (0.41 nonsynonymous mutations per Mb, on average). It positively correlated with age, recurrence risk, and the distant Metastasis, patient Age, Completeness of resection, local Invasion, and tumor Size (MACIS) score. These results may reflect an indolent nature of the majority PTC cases on the one hand, and on the other hand, represent the molecular basis of aggressive PTC. Until recently, the *BRAF* V600E mutation was considered a marker of poor prognosis in PTC. Many studies were pointing to a possible relationship between its presence and the worse outcome [5,6]. Nevertheless, its role as a prognostic or predictive factor is still controversial [7]. The frequency of the *BRAF* V600E mutation ranges from 23 to 83% in different PTC cohorts. Its presence was demonstrated in both classic PTCs as well as in its aggressive subtypes [8]. According to the current ATA guidelines for diagnostics and treatment of differentiated thyroid carcinoma (DTC), the *BRAF* V600E mutation may be considered in risk stratification, however, in the context of clinical data only [9]. 

In 2013 two somatic mutations of telomerase reverse transcriptase (*TERT*) gene promoter were discovered in different solid cancers, including TCs [10,11,12]. Two alterations, c.-124C > T (C228T) and c.-146C > T (C250T) were reported in PTC with the prevalence of 11.3% [13]. Their frequency was higher in more aggressive histopathological PTC variants [10]. *TERT* encodes the catalytic subunit of telomerase, responsible for telomeres maintenance at the end of chromosomes. Telomerase, typically not expressed in somatic cells, is reactivated in cancer cells. It protects telomeres from further shortening and is also involved in cancer cell proliferation, resistance to apoptosis and antigrowth signals, increased angiogenesis, and metastatic potential [14]. It has been demonstrated that C228T and C250T *TERT* promoter (*TERT*p) mutations were much more prevalent in PTCs harboring *BRAF* V600E mutation. This co-occurrence was related to poor prognostic factors, including tumor size, older age at diagnosis, distant metastases, or shorter progression-free survival [13,15,16]. Also, the co-existence of *RAS* and *TERT*p mutations was associated with a worse prognosis. Shen et al. proposed a six-genotype genetic prognostic model for PTC, dividing patients into the following risk groups regarding the detected mutation: *BRAF* V600E/*RAS* mutation and *TERT*p mutation  >>>> *BRAF* V600E  = *TERT*p mutation alone  > *RAS* mutation alone  =  wild-type genes [17]. 

Since the discovery of *TERT*p mutations in thyroid cancers, most studies have focused on their clinical implications. However, the data concerning their molecular consequences and potential differences/similarities to *BRAF* V600E-positive TCs are scarce. To our best knowledge, there was only one published study devoted to the transcriptomic analysis of *TERT*p-mutated PTCs [18]. Therefore, in the present study, we focused on the gene expression profile of *TERT*p-mutated PTC in BRAF(+) cases. We aimed to analyze the molecular consequences of *TERT*p alterations that could explain their impact on PTC outcomes. As our PTC cohort involved mainly patients showing unfavorable clinical course, we tried to find molecular mechanisms, responsible for PTC aggressiveness.

## 2. Results

### 2.1. Mutational Analysis for BRAFV600, TERTp, and RAS Somatic Mutations and Their Association with Histopathological and Clinical PTC Features

Thirty-two out of 54 PTC cases (59%) were positive for at least one of the analyzed genetic alterations. Initially, using the Sanger sequencing method, the *BRAF* V600E mutation was detected in 24 PTC samples. However, using the QPCR technique, enabled us to identify this genetic alteration in the other 3 PTC cases. Finally, 27/54 (50%) PTCs were BRAF(+). In nine out of 27 (33%) PTCs harboring *BRAF* V600E mutation, additional somatic alterations were detected: eight PTCs were positive for *TERT*p mutations (six cases with C228T and two with C250T mutation), and one PTC was positive for *NRAS* mutation. In a further five cases, mutations in *RAS* genes were found (two PTCs with *HRAS* mutations and three PTCs with *NRAS* mutations). However, we did not observe the co-existence of *RAS* and *TERT*p mutation. In the remaining 22 PTCs, no mutation was detected (NM-PTC). Considering distinct clinical and histopathological PTC features, the presence of *BRAF*, *RAS,* and *TERT*p mutations did not show any association with sex, histopathological PTC variant, multifocality, invasion of the tumor capsule, angioinvasion, or the presence of lymph node metastases (Table 1). In contrast, *TERT*p mutations (comparison between BRAF(+)TERTp(+) and BRAF(+)TERTP(−) PTCs) were significantly associated with tumor diameter, invasion of surrounding tissues, pN1b feature, the locally persistent disease after the 1st surgery, and distant metastases.

### 2.2. Impact of Studied Mutations on Gene Expression Profile of PTCs

**Figure 1:**
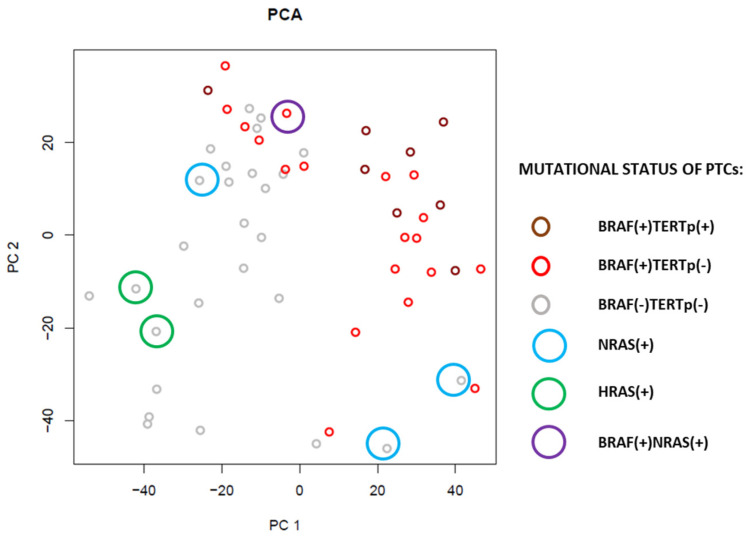
PCA analysis of PTCs with reference to *BRAF*, *TERT*p and *RAS* mutational status.

Principal Component Analysis (PCA) was performed in order to explore major sources of gene expression variation. In the first and second components, the main source of variation was the presence of the *BRAF* V600E mutation (Figure 1). The strongest similarity was observed between BRAF(+)TERTp(+) and BRAF(+)TERTp(−) PTCs. PTCs harboring *HRAS* and *NRAS* mutations, in turn, were grouped with NM-PTCs. However, they were not grouped as close as BRAF(+) cases.

### 2.3. Mutational Status and TDS, and BRS Coefficients

**Figure 2:**
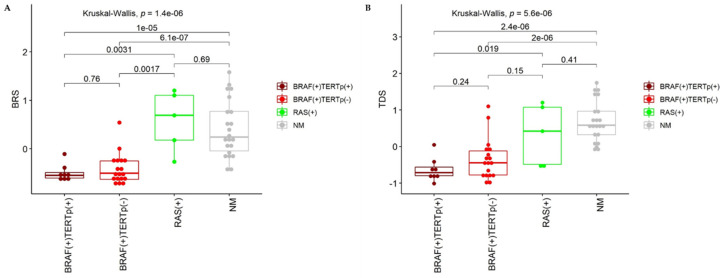
Analysis of BRS (**A**) and TDS (**B**). High BRS depicts RAS-like, whereas low BRS depicts BRAF-like samples. High TDS depicts high expression of thyroid specific genes and high thyroid differentiation. TDS: The Thyroid Differentiation Score, BRS: the BRAF-like RAS-like Score. *p*-values shown on plots were calculated with Kruskal-Wallis and two-tailed Mann-Whitney tests.

We calculated the Thyroid Differentiation Score (TDS) and the BRAF-like RAS-like Score (BRS). BRS is a gene expression score that indicates whether a sample is more similar to samples harboring the *BRAF* V600E mutation (BRAF-like samples, low BRS score) or *RAS* mutation (RAS-like samples; high BRS score) (Figure 2). As expected, in our study, samples harboring the *BRAF* V600E mutation were more BRAF-like, whereas samples harboring *RAS* mutation were more RAS-like. The difference in BRS score between BRAF(+) and RAS(+) samples was statistically significant. NM-PTC samples were more RAS-like, and their BRS was significantly higher than in BRAF(+) samples (Figure 2A). However, the separation between NM-PTCs and BRAF(+) samples was not perfect. It may suggest that some of the samples without *BRAF*, *TERT*p, and *RAS* mutation, harbored other genetic alterations associated with gene expression profile similar to BRAF(+) PTCs. 

TDS analysis showed significant downregulation of thyroid-specific genes in PTCs harboring *BRAF* and *TERT*p mutation comparing to cases without these alterations (RAS(+) PTCs and NM-PTCs; Figure 2B). However, there was no statistically significant difference in TDS between BRAF(+)TERTp(+) and BRAF(+)TERTp(−) PTCs. Also, no statistically significant difference was observed between RAS(+) PTC cases and BRAF(+)TERTp(−) PTCs or NM-PTCs.

### 2.4. TERTp Mutations and Gene Expression Profile of PTCs

**Figure 3:**
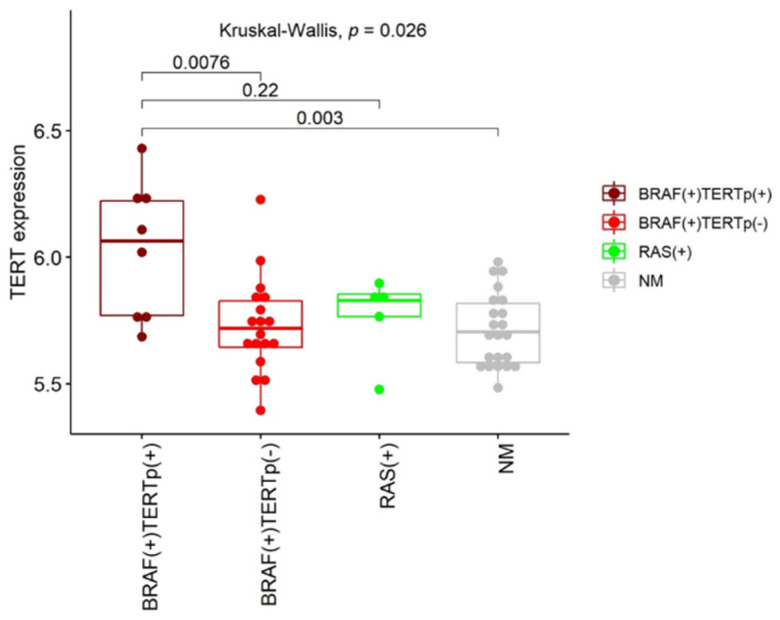
*TERT* gene expression in PTC sets with different mutations; RAS(+) cohort was BRAF(−) and TERTp(−). *p*-values shown on plot were calculated with Kruskal-Wallis and two-tailed Mann-Whitney tests.

Our study mainly focused on the impact of *TERT*p mutations on gene expression profile. However, the presence of other somatic mutations was also considered. First, we analyzed *TERT* gene expression depending on the presence/absence of mutations within its promoter region. We observed a tendency of *TERT* up-regulation in PTCs harboring C228T or C250T mutation with significant differences limited to the following comparisons: BRAF(+)TERTp(+) vs. BRAF(+)TERTp(−) PTCs and BRAF(+)TERTp(+) vs. NM-PTCs (*p* = 0.0076 and *p* = 0.003, respectively; Figure 3). However, changes in the *TERT* gene expression between TERTp(+) and RAS(+) PTCs did not reach statistical significance (*p* = 0.22). Next, several comparative analyses were performed to find differences in gene expression that could result from the presence of a particular genetic alteration (*BRAF*, *TERT*p, or *RAS* mutations; Table 2). The highest number of significantly differentiating genes was obtained when PTCs harboring any of the studied mutations were compared to NM-PTCs (2543 genes; FDR < 0.05). Comparison of BRAF(+) PTCs with RAS(+) PTCs resulted in 709 differentially expressed genes (FDR < 0.05), whereas only four genes showed significant differences in their expression between BRAF(+)TERTp(+) and BRAF(+)TERTp(-) cases (FDR < 0.05) (Table 3). A small number of significantly changed genes was also found when RAS(+) PTCs and NM-PTCs were compared (3 genes; FDR < 0.05). The lists of all genes are given in Table S1.

**Figure 4:**
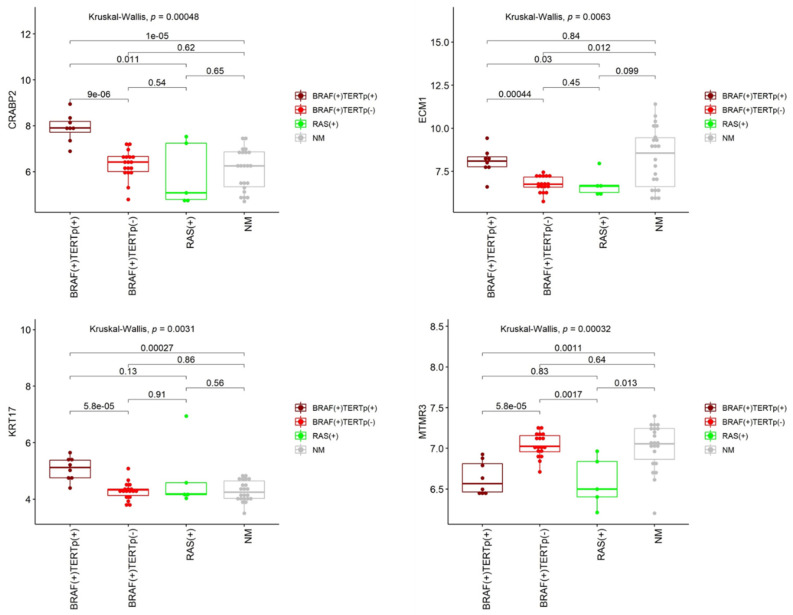
Expression of four genes significantly differentiating BRAF(+)TERTp(+) and BRAF(+)TERTp(−) PTCs in all analyzed PTC cases; RAS(+) cohort was BRAF(−) and TERTp(−). *p*-values shown on plots were calculated with Kruskal-Wallis and two-tailed Mann-Whitney tests.

Of the four genes (*CRABP2*, *ECM1, KRT17*, and *MTMR3*) differentially expressed between TERTp(+) and TERTp(−) PTCs, all but one, *MTMR3*, were up-regulated in PTCs harboring *TERT*p mutation (Table 3; Figure 4). Besides, the *MTMR3* gene showed significantly higher expression in BRAF(+)TERTp(−) PTCs comparing to RAS(+) ones. The *ECM1* gene, in turn, was down-regulated in mutated PTCs in comparison to NM-PTCs.

### 2.5. Validation of TERTp-Dependent Genes

Validation of genes differentially expressed between TERTp(+) and TERTp(−) PTCs was performed on an independent PTC cohort, made available by the TCGA study [4]. In this analysis, two sets were considered: BRAF(+)TERTp(+) and BRAF(+)TERTp(−) PTCs (28 and 207 PTCs, respectively; with the exclusion of all PTCs harboring *RAS* mutations). Only one out of four genes studied, the *CRABP2* gene was confirmed as significantly differentiating BRAF(+) PTCs with and without *TERT*p mutation (Table 4).

### 2.6. Signaling Pathways and Gene Ontology Analysis

To evaluate potential differences in signaling pathways and changes in gene ontology (GO) terms resulting from the presence of *TERT*p mutations, the Gene Set Enrichment Analysis (GSEA) was used. Comparing BRAF(+)TERTp(+) and BRAF(+)TERTp(-) samples, we obtained 39 significantly enriched KEGG pathways, 644 significantly enriched GO Biological Process (BP) terms, 131 significantly enriched GO Cellular Component (CC) terms, and 209 significantly enriched GO Molecular Function (MF) terms (FDR < 0.05). Table 5 presents ten significantly enriched gene groups with the highest absolute normalized enrichment score (NES) for each analysis (Table 5).

Among significantly enriched (FDR < 0.05) gene groups, six groups were related to telomeres (Table 6).

## 3. Discussion

The presence of the *TERT*p mutation resulted in a changed expression of four genes only. It is the most important result of our analysis of the PTC gene expression profile. Moreover, only one of these genes was confirmed in an independent set of PTC samples made available by the TCGA [4] consortium. However, one should notice that the TCGA cohort consisted mainly of low-risk PTCs. 

One of the hallmarks of malignant neoplasms is an evasion of replicative senescence and out-of-control proliferation, leading to immortalization of cancer cells [19]. The pivotal mechanism underlying this process is the reactivation of telomerase, an enzyme typically not expressed in somatic cells, but activated in about 80–90% of all malignant neoplasms [20]. Telomerase is a complex protein. However, its core is composed only of catalytic component TERT and internal telomerase RNA template (TERC) [21]. TERC is ubiquitously expressed in various human cells. On the contrary, TERT is repressed in somatic cells leading to telomerase silencing [22]. Its induction/telomerase activation not only provides telomeres stabilization but is also related to several oncogenic processes, independent of telomeres lengthening. For instance, it has been shown that TERT directly interacts with β-catenin and, as a consequence, stimulates epithelial-mesenchymal transition (EMT) and stemness of cancer cells, and, by interaction with NF-κB p65, up-regulates the expression of metalloproteinases (MMPs) in cancer cells [23,24]. Moreover, TERT contributes to survival signaling, growth signaling, invasion/metastasis, angiogenesis, DNA methylation, genetic aberrations, and even to radio/chemo-resistance, which make TERT an important factor related to a higher aggressiveness of cancer cells [22]. Also, in thyroid cancer, TERT induction has been linked to a poorer prognosis, higher risk of metastases, recurrence, and even death [13]. There are several mechanisms underlying *TERT* activation with *TERT*p somatic mutations being the most widely studied in many cancer types since their discovery in 2013 [11,12]. In TCs, their frequency is considered as intermediate. However, it increases dramatically from microcarcinomas (frequency reported at the level of about 5%) [25] to aggressive poorly differentiated and anaplastic TCs (48.8% and 41.8%, respectively) [20]. It seems to be more common in TCs harboring *BRAF* V600E mutation [26]. Although a lot of data concerning the clinical significance of *TERT*p mutations in TCs has been published, there is still much to discover in the field of their molecular consequences. To our best knowledge, this is the first study that analyses the impact of *TERT*p mutations on PTC transcriptome in BRAF(+) samples. 

Our previous study showed that in Polish PTC patients, *TERT*p mutations are present in 8.5% of cases [16]. However, in the current analysis, in which almost half of the PTC cases presented distant metastases, the frequency of C228T and C250T mutations increased up to 14.8%. Of 30 non-metastatic cases, two PTCs were TERTp(+) (6.7%), whereas six out of 24 PTCs with distant metastases harbored a *TERT*p mutation (25%). Similar data obtained Gandolfi et al., who showed the presence of these alterations in 10% of non-metastatic PTCs and 33% of PTCs with distant metastases [27]. All PTCs harboring *TERT*p mutation in our study were *BRAF* V600E-positive. Moreover, in one PTC with *BRAF* mutation, *NRAS* point mutation was reported. However, none of the studied cases displayed the co-existence of *TERT*p and *RAS* somatic mutations. 

In the current study, we focused mainly on genes and processes that are significantly altered in the presence of *TERT*p mutations. As indicated in previous studies, *TERT*p mutations were associated with elevated *TERT* expression [28] and, as presented by Fredriksson et al., this association was exceptional in its strength and was highest in copy number-stable cancers such as thyroid carcinoma [29]. Our results are in agreement with these data. We observed significant up-regulation of *TERT* transcript in TERTp(+) PTCs comparing to TERTp(−) ones harboring *BRAF* V600E mutation and NM-PTCs. Although there was no significant difference in *TERT* expression between TERTp(+) and RAS(+) PTCs, a tendency of *TERT*p mutations-dependent up-regulation of *TERT* mRNA was visible. However, a limited number of RAS(+) PTCs might impair the results and statistics. 

The next step was a closer look at the whole gene expression profiles of our PTC set with a particular focus on cases with *TERT*p mutation. There was no surprise that all PTCs harboring *BRAF* mutation grouped close to each other in an unsupervised PCA analysis. It was the most potent differentiating factor within the first and second components. RAS(+) PTCs grouped with NM-PTC cases. PCA did not show differences between BRAF(+)TERTp(+) and BRAF(+)TERTp(−) samples. Moreover, according to the BRS, BRAF(+) PTCs, including all TERTp(+) cases, were BRAF-like and most RAS(+) and NM-PTCs were RAS-like. However, one RAS(+) and several NM-PTCs were more similar to BRAF-like PTCs. It might result from the presence of some additional alterations, and, in consequence, their gene expression profile was more similar to BRAF(+) PTCs. Some of the NM-PTCs may harbor *RET* fusions. In the TCGA study, *RET* rearrangements were present in 6.8% PTCs (33/484), and nearly all of them were weakly BRAF-like. However, we focused mainly on *TERT*p mutations and genetic alterations that most commonly co-exist with them. The presence of *TERT*p mutations, in turn, may impair the expression of thyroid-specific genes by down-regulating them. Although there were no differences in TDS among BRAF(+) PTCs with and without *TERT*p mutations, this association was significant in comparison of BRAF(+)TERTp(+) cases to RAS(+) and NM-PTCs. These data suggest that *TERT*p mutations may be crucial in PTC dedifferentiation and aggressiveness. It is supported by the association of *TERT*p mutations with poor prognostic factors, observed in our PTC cohort. Larger tumor size, invasion of the surrounding tissues, the locally persistent disease after the first surgery, pN1b, and distant metastases were significantly associated with mutated *TERT*p, which is in concordance with previous studies [13].

Taking into consideration significant diversity of BRAF-like PTCs, noticed in TCGA study [4] and a higher frequency of *TERT*p mutations in PTCs harboring *BRAF* V600E alteration [30,31], we focused on possible gene expression differences within BRAF(+) PTCs resulting from the presence of *TERT*p mutations. The observed difference was subtle. However, we did not expect any differences since all TERTp(+) cases were simultaneously BRAF(+). We are aware that not all BRAF(+) PTCs show poor outcomes, so there must be some additional factors responsible for disease aggressiveness. *TERT*p mutations certainly participate in this process. We obtained four genes differentiating BRAF(+)TERTp(+) PTCs and BRAF(+)TERTp(−) ones (*CRABP2*, *ECM1, KRT17,* and *MTMR3*). One out of these four genes, the *CRABP2* gene, was positively validated on an independent PTC cohort, although with a lower number of high-risk cases. This result confirms that the TERTp(+)-dependent difference exists. The *CRABP2* gene encodes the cellular retinoic acid-binding protein 2 that is responsible for retinoic acid (RA) transport to retinoic acid receptors (RARs) within the nucleus, inhibiting cell growth and proliferation [32]. That is why *CRABP2* is considered as an antitumor agent. However, there are contradicting data suggesting the necessity of further studies on the *CRABP2* role in tumorigenesis. For instance, CRABP2 protein was identified as a subtype-specific biomarker of ovarian cancer, since its expression positively correlated with tumor grade and cancer stage [33]. Similarly, higher CRABP2 expression corresponded to invasive retinoblastomas [34] and poorer prognosis in estrogen receptor-negative breast cancer [35]. Moreover, its overexpression was suggested to be a late event of pancreatic carcinogenesis that could be used as a marker to distinguish pancreatic ductal adenocarcinomas from other benign pancreatic conditions [36]. Kim et al. proposed plasma CRABP2 as a novel diagnostic and prognostic marker in non-small cell lung cancer [37]. In our study, the expression of *CRABP2* was three times higher in the presence of *TERT*p mutation (in TCGA data FC was 2.2). Together with previous studies, it may support the hypothesis that an elevated *CRABP2* expression is a late event, characteristic for more advanced and aggressive tumors. These data do not confirm the recognized main role of CRABP2 as an antitumor agent. An alternative mechanism of CRABP2 activity has been proposed. It has been proven that CRABP2 mediated proliferative activity not by interacting with RAR, but through PPARbeta/delta receptor in the presence of fatty acid binding protein 5 [32]. 

Two genes, *KRT17* and *ECM1*, which expression was elevated in our TERTp(+) PTCs, demonstrated higher expression in a variety of cancer types. Keratins are components of the cytoskeleton and play a major role in cell protection and structural support. Keratin KRT17, belonging to type I keratin, was regenerated and highly expressed in many cancers [38], including gastric cancer [39], cervical cancer [40], oral squamous cell carcinoma [41], and breast cancer [42]. Moreover, KRT17 expression was proposed as a prognostic marker that can discriminate postoperative stage II patients with colorectal cancer with a high probability of disease recurrence, as support to available risk stratification factors [43]. Extracellular matrix protein 1 (ECM1), in turn, is a glycoprotein involved in cell proliferation, angiogenesis, migration, and metastases. Its elevated expression was observed in several malignancies, including thyroid, gastric, colorectal, lung carcinoma, invasive ductal breast carcinomas, hepatocellular cancer, and others [44,45]. A significantly high increase in *ECM1* expression was observed in malignant epithelial tumors, especially in these tumors with distant metastases [44]. This observation would explain higher *ECM1* expression in our TERTp(+) PTCs when compared to BRAF(+)TERTp(-) and RAS(+) PTCs. However, *ECM1* occurred to be down-regulated in mutated PTCs in comparison to NM-PTCs. So, its role as a potential marker of TERTp(+) PTCs seems questionable. 

Regarding the 4-gene signature of TERTp(+) PTCs in our study, the *MTMR3* gene (encodes myotubularin-related protein 3) was the only down-regulated gene in TERTp(+) PTCs comparing to TERTp(−) ones. MTMR3 belongs to phosphoinositide 3-phosphatases with affinity to Ptdlns5P [46]. It may impair or enhance tumor growth in different types of cancer. For example, the lack of MTMR3 was shown to repress the proliferative and invasive potential of oral cancer cells [47]. Its exogenous expression inhibited the growth of transfected lung carcinoma cells [48]. On the contrary, its higher expression had negative effects on overall survival and relapse-free survival in patients with breast cancer [49]. Nevertheless, the role of *MTMR3* in TERTp(+) PTCs requires further studies.

Our signaling pathways analysis revealed 39 enriched KEGG pathways differentially changed between BRAF(+)TERTp(+) and BRAF(+)TERTp(−) PTCs. The majority of pathways with the highest absolute NES (normalized enrichment score) value were enriched by down-regulated genes, among them Inositol phosphate metabolism, Phosphatidylinositol signaling system, and Ubiquitin mediated proteolysis pathways. The critical feature of cancer cells differentiating them from healthy ones is related to the reprogramming of fundamental pathways determining distinct processes, including proliferation, differentiation, and motility. The Ubiquitin pathway plays an essential role in the regulation of cell growth and cell proliferation through the control of key cell cycle proteins. Because of its involvement in crucial biochemical processes, it is a potential target for cancer-related deregulation. Impaired proteolysis of cell cycle regulators was reported in many human cancers as being contributed to tumorigenesis [50,51]. Phosphatidylinositol signaling system is also known as associated with cancer, mainly by its cooperation with the PI3K-Akt pathway that mediates cell proliferation, survival, and metabolism. Mainly, PI3K and PTEN play a key role in cancer, but also other members of this pathway seem to be implicated in the progression of tumors [52]. 

Among pathways with the highest NES value, enriched by up-regulated genes, we found the Neuroactive ligand-receptor interaction pathway, which includes receptors and ligands associated with intracellular and extracellular signaling pathways involved in the progression of the bladder, renal cell, and prostate cancer [53,54,55]. Up-regulated genes also enriched the Olfactory transduction pathway. Olfactory receptors (OR), playing a crucial role in healthy tissues, were also involved in multiple pathological processes, including hepatocarcinoma, non-small cell lung cancer, colorectal cancer, melanoma, and breast cancer [56]. Some of these ORs were proposed as markers in different cancer types to discriminate between cancer and healthy tissues. Moreover, comparing BRAF(+)TERTp(+) and BRAF(+)TERTp(−) PTCs, we found six significantly enriched gene groups related to telomeres. All of these gene groups had a negative NES value (i.e., they are enriched in genes downregulated in BRAF(+)TERTp(+) cases), which may suggest that the presence of *TERT*p mutations impairs processes involved in telomere maintenance. 

Difference in gene expression profile found in our BRAF(+) PTCs as dependent on the mutated *TERT* promoter was subtle. However, positive verification of one out of four differentiating genes on an independent PTC cohort allows to hypothesize that obtained data will still be valid on a much larger tumor set. Especially since our and validation PTC sets differed in two important features- number of high-risk PTCs, that was incomparably larger in our cohort, and presence of *TERT*p mutations only as co-existing with *BRAF* V600E mutation in our PTC set. Despite these differences expression of *CRABP2* gene was significantly up-regulated in *TERT*p(+) cases in both cohorts.

So far, there was only one published study that analyzed the impact of the presence of *TERT*p mutations on PTC transcriptome [18]. Chien et al., who used the data obtained from the TCGA study [4], found *TERT*p mutations to be associated with proliferative and metabolic alterations in PTC. Pathways related to DNA damage response and cell cycle regulation were enriched among up-regulated genes, whereas transporter and metabolic activities were overrepresented among down-regulated genes [18]. Although they analyzed a much larger group, they found no association between *TERT*p mutations and *BRAF* genetic alterations. No difference regarding thyroid differentiation genes was observed. Our PTC cohort was smaller. All TERTp(+) PTCs were BRAF(+). The main limitation of this study is related to the coexistence of the *BRAF* mutation in all TERTp(+) PTC samples. However, the presence of *TERT*p mutations, without co-existing *BRAF* or *RAS* genetic alterations is rare in PTC. In our previous study, 3 out of 189 PTCs (1.6%) were positive only for *TERT*p mutation (no *BRAF* and no *RAS* mutations detected). We found significantly lower TDS value in TERTp(+) PTCs comparing to RAS(+) and NM-PTCs. However, the limited number of samples and the presence of other than *TERT*p mutations in our analysis impaired the obtained results. In addition, because of the selection bias, almost half of our PTC patients had distant metastases. It increased the number of TERTp(+) PTCs in our cohort. It is also the crucial difference between our and TCGA PTC cohorts since the latter one included mainly low-risk PTCs (only 8 PTCs had distant metastases) [4]. Moreover, the use of a larger set of high-risk PTCs confined our cohort to cases with co-existing *BRAF* and *TERT*p mutations and obligated us to search for differences mainly within the BRAF(+) PTC set. On the one hand, it is a limitation of our analysis. On the other hand, it is important to find mechanisms responsible for PTC aggressiveness, since the *BRAF* V600E mutation as a prognostic marker in PTC is still controversial and is considered in treatment or risk stratification only regarding clinical features. 

Despite mentioned limitations of the study, there are clear differences in gene expression profile between BRAF(+) PTC tumors carrying *TERT*p mutation and PTC tumors with only *BRAF* mutation. This suggests potential role of *TERT*p mutations in down-regulation of thyroid specific genes. Additionally, we confirmed that mutated *TERT* promoter is associated with poor prognosis of PTC and it might have a potential value as a prognostic marker.

## 4. Materials and Methods 

### 4.1. Patients Characteristics

The study cohort consisted of 54 PTC cases, operated at Maria Sklodowska-Curie National Research Institute of Oncology Gliwice Branch, in Gliwice, Poland. The patients were selected based on their clinical course to obtain representative groups with and without distant metastases. The ‘metastatic cohort’ involved 24 PTC cases. The presence of distant metastases was confirmed by X-ray, computed tomography, scintigraphy, and/or histopathological examination. The ‘control group’, consisted of PTC patients without distant metastases. We intended to match the groups for gender, age, and primary advancement. However, the exact matching was not possible. The mean follow-up was 95.9 months (78.6–112.7 months). Clinical and histopathological characteristics are summarized in Table 1, together with the mutational status, given in the “Results” section. 

The use of human tissue was approved by the Bioethics Committee at Maria Sklodowska-Curie National Research Institute of Oncology Gliwice Branch (Committee decision from 25 September 2001). Written informed consent to analyze the tissue was obtained from all patients. All clinical data were anonymized and de-identified before the analysis.

### 4.2. BRAF V600E and TERTp Mutation Analysis

DNA was isolated from the resected thyroids with the DNeasy Blood & Tissue Kit (Qiagen, GmbH, Hilden, Germany) according to the manufacturer protocol. Both *BRAF* and *TERT*p mutations were analyzed by PCR amplification and direct sequencing of the hot spots (V600E and C228T, C250T, respectively). A 160-bp region of the *BRAF* 15th exon, containing the codon 600, was amplified using following primers set: forward-5′-tgttttcctttacttactacacctca-3′; reverse-5′- gcctcaattcttaccatcca-3′, whereas the amplification of the 157-bp fragment of the *TERT*p containing C228T and C250T localizations was performed with the primers: forward-5′-ccccttcaccttccagctc-3′; reverse-5′-cagcgctgcctgaaactc-3′. The PCR steps were as follows: initial denaturation at 95 °C for 15 min, 34 cycles of denaturation at 95 °C for 30 s; primers annealing at 56.5 °C and 59.7 °C (for *BRAF* and *TERT*p, respectively) for 30 s; elongation at 72 °C for 30 s and the final elongation at 72 °C for 5 min. The PCR products were checked for the quality with gel electrophoresis, purified with enzymes, alkaline phosphatase (SAP) and exonuclease I (Life Technologies, Carlsbad, CA, USA), and then subjected to standard sequencing with ABI PRISM^TM^ 1.1 BigDye Terminator Cycle Sequencing Ready Reaction Kit (Life Technologies) on the 3130xl Genetic Analyzer Applied Biosystems (Life Technologies). All samples were also analyzed for *BRAF* V600E mutations with the THDNA-RT64 kit (EntroGen, Woodland Hills, CA, USA). For the more detailed description of this method, follow the next subsection entitled “*RAS* mutations detection”.

### 4.3. RAS Mutations Detection

Quantitative real-time PCR (QPCR) was used in order to analyze *RAS* somatic mutations. The analysis was performed with the THDNA-RT64 kit (EntroGen) on the QuantStidio 12K Flex Real-Time PCR System (Thermo Fisher Scientific, Waltham, MA, USA), according to the manufacturer protocols. This test is designed to analyze 15 most frequent somatic point mutations in PTCs within the *RAS* genes and V600E mutation in the *BRAF* gene. It provides information about whether there is a mutation in *HRAS*, *NRAS*, *KRAS,* or not, without pointing which mutation it is. The exact list of mutations analyzed with this test is given in Table S2. 

### 4.4. Microarray Analysis

The GeneChip Human Gene 1.0 arrays (Affymetrix, Santa Clara, CA, USA) were used. The detailed methodology was described in our previous work [57]. 

Background correction, normalization and probe set summarization were done using the Robust Multichip Average (RMA) algorithm with library oligo v1.48.0 from R v3.6.0 environment, and custom CDF files from BrainArray (ENTREZG; v24) [58,59,60]. Principal component analysis (PCA) was performed with R/Bioconductor. 

The selection of differentially expressed genes was performed using the limma 3.40.6 library [61]. Gene set analysis was performed using Gene-Set Enrichment Analysis (GSEA) implemented in the clusterProfiler 3.12.0 library [62,63]. The t-statistic was used as a gene ranking metric, and 10,000 gene set permutations were performed to calculate p-values and Normalized Enrichment Scores (NES). Pathways from the Kyoto Encyclopedia of Genes and Genomes (KEGG) and terms from the Gene Ontology (GO) with minimal gene size of 10 and maximal of 600 were used in GSEA [64,65]. 

All p-values were corrected for false discovery rate (FDR), according to Benjamini and Hochberg [66]. Corrected *p*-values < 0.05 were considered statistically significant.

### 4.5. TCGA Data Analysis

Gene expression data for TCGA PTC samples (based on RNA-seq data) were retrieved using the TCGAbiolinks 2.12.6 library [67]. The comparison was performed between 28 BRAF(+)TERTp(+) samples and 207 BRAF(+)TERTp(−) (only RAS-negative samples were analyzed) using edgeR 3.26.8 embedded in the TCGAbiolinks library [68]. The read counts were modeled using the negative binomial generalized log-linear model, and a likelihood ratio test was used to assess statistical significance [69]. *p*-values were corrected for false discovery rate (FDR), according to Benjamini and Hochberg [66]. Corrected *p*-values < 0.05 were considered statistically significant.

### 4.6. Calculation of Expression Scores

For each microarray sample, two scores based on gene expression were calculated: *BRAF* V600E-*RAS* Score (BRS) and Thyroid Differentiation Score (TDS) [4]. 

BRS is a 71-gene signature, developed in TCGA study [4], which quantifies the extent to which the gene expression profile of a given tumor resembles either the *BRAF* V600E or *RAS* mutant profiles. According to TCGA study, it comprises of 71 genes (13 genes up-regulated in *RAS* mutant samples and 58 genes up-regulated in *BRAF* V600E mutant samples). In our paper, BRS was calculated in three steps: (1) we calculated sum of expression of 13 genes up-regulated in *RAS* mutant samples minus the sum of expression of 58 genes up-regulated in *BRAF* V600E mutant samples; (2) we divided obtained measures by the number of genes in signature; (3) finally we centered obtained values at the mean across samples. Tumors with low BRS were defined BRAF V600E-like, while tumors with high BRS were defined as RAS-like.

TDS is a summarized expression level of 16 thyroid metabolism and function genes, developed in the TCGA study [4]. The expression values of each gene were first centered at the median across samples. Then, the mean across the 16 genes was calculated for each sample. Higher TDS depicts higher differentiation of the tumor.

### 4.7. Statistical Analysis of Clinico-Histopahological Data

Categorical data were summarized with numbers and percentages. Continuous data were summarized with medians and interquartile ranges. Comparisons of categorical variables were performed using Fisher’s exact test. Comparisons of continuous variables were performed using two-tailed Mann-Whitney U test (in case of two groups comparisons), and Kruskal-Wallis rank sum test (in case of comparisons of more than two groups). *p* values < 0.05 were considered statistically significant. Statistical analyses were performed using the R software version 3.6.0 and Gmisc package version 1.9.0 [70,71].

## 5. Conclusions

We found four genes differentiating TERTp(+) and TERTp(−) PTCs harboring *BRAF* mutation. It is a small but clear difference, since one of these genes, *CRABP2*, was validated on an independent set of PTCs. However, their potential use in diagnostics or risk stratification requires additional prospective studies on larger PTC cohorts. Our study is the first one in which the TERTp-dependent PTC gene expression profile was analyzed in *BRAF* V600E positive samples only. *TERT*p mutations may be associated with down-regulation of thyroid-specific genes, with their expression being significantly lower comparing to RAS(+) and NM-PTCs. *TERT*p mutations are related to higher PTC aggressiveness. These are not a frequent molecular event in PTCs, however, if present, always associated with poorer prognosis and a higher level of molecular aberrations.

## Figures and Tables

**Table 1:** Characteristics of analyzed PTC cohort with *BRAF*, *RAS* and *TERT*p mutational status, and associations between clinico-histopathological and molecular features.

Clinical/Histopathological Features	Total	NM-PTC	RAS(+)	BRAF(+)RAS(+)	BRAF(+)TERTp(−)	BRAF(+)TERTp(+)	Comparison Between the 5 Mutational Subgroups *p*-Value	BRAF(+)TERTp(+) vs. BRAF(+)TERTp(−) *p*-Value
	No. 54	No. 22	No. 5	No. 1	No. 18	No. 8		
**Sex**							0.94	0.66
Male	21 (38.9%)	9 (40.9%)	2 (40.0%)	0 (0.0%)	6 (33.3%)	4 (50.0%)		
Female	33 (61.1%)	13 (59.1%)	3 (60.0%)	1 (100.0%)	12 (66.7%)	4 (50.0%)		
**Age at diagnosis**							0.005	0.14
	48.5 (31.2–68.2)	33.0 (21.0–46.8)	49.0 (49.0–57.0)	17.0 (17.0–17.0)	55.0 (42.5–72.2)	72.5 (56.5–77.2)		
**Classification according to the age at diagnosis**							0.0005	0.28
<45 years	19 (35.2%)	14 (63.6%)	0 (0.0%)	1 (100.0%)	4 (22.2%)	0 (0.0%)		
>45 years	35 (64.8%)	8 (36.4%)	5 (100.0%)	0 (0.0%)	14 (77.8%)	8 (100.0%)		
**Histopathological PTC classification**							0.15	1.00
Classical variant	46 (85.2%)	17 (77.3%)	3 (60.0%)	1 (100.0%)	17 (94.4%)	8 (100.0%)		
Follicular variant	8 (14.8%)	5 (22.7%)	2 (40.0%)	0 (0.0%)	1 (5.6%)	0 (0.0%)		
**Tumor diameter [mm]**							0.014	0.0008
	24.0 (15.0–30.0)	21.0 (13.5–30.0)	30.0 (12.0–35.0)	31.0 (31.0–31.0)	18.0 (13.5–25.0)	40.0 (29.5–61.2)		
**Multifocality**							0.59	0.40
No	26 (48.1%)	9 (40.9%)	2 (40.0%)	1 (100.0%)	11 (61.1%)	3 (37.5%)		
Yes	28 (51.9%)	13 (59.1%)	3 (60.0%)	0 (0.0%)	7 (38.9%)	5 (62.5%)		
**Invasion of the tumor capsule**							0.17	0.22
No	25 (46.3%)	8 (36.4%)	4 (80.0%)	1 (100.0%)	10 (55.6%)	2 (25.0%)		
Yes	29 (53.7%)	14 (63.6%)	1 (20.0%)	0 (0.0%)	8 (44.4%)	6 (75.0%)		
**Invasion of the surrounding tissues**							0.041	0.014
No	41 (75.9%)	16 (72.7%)	5 (100.0%)	1 (100.0%)	16 (88.9%)	3 (37.5%)		
Yes	13 (24.1%)	6 (27.3%)	0 (0.0%)	0 (0.0%)	2 (11.1%)	5 (62.5%)		
**Angioinvasion**							0.27	0.31
No	48 (88.9%)	18 (81.8%)	4 (80.0%)	1 (100.0%)	18 (100.0%)	7 (87.5%)		
Yes	6 (11.1%)	4 (18.2%)	1 (20.0%)	0 (0.0%)	0 (0.0%)	1 (12.5%)		
**pN1a**							0.25	0.20
No	22 (40.7%)	7 (31.8%)	2 (40.0%)	0 (0.0%)	11 (61.1%)	2 (25.0%)		
Yes	32 (59.3%)	15 (68.2%)	3 (60.0%)	1 (100.0%)	7 (38.9%)	6 (75.0%)		
**pN1b**							0.095	0.038
No	27 (50.0%)	9 (40.9%)	3 (60.0%)	0 (0.0%)	13 (72.2%)	2 (25.0%)		
Yes	27 (50.0%)	13 (59.1%)	2 (40.0%)	1 (100.0%)	5 (27.8%)	6 (75.0%)		
**Locally persistent disease after the 1st surgery**							0.012	0.005
No	47 (87.0%)	19 (86.4%)	5 (100.0%)	1 (100.0%)	18 (100.0%)	4 (50.0%)		
Yes	7 (13.0%)	3 (13.6%)	0 (0.0%)	0 (0.0%)	0 (0.0%)	4 (50.0%)		
**Local recurrence**							0.01	0.086
No	49 (90.7%)	21 (95.5%)	4 (80.0%)	0 (0.0%)	18 (100.0%)	6 (75.0%)		
Yes	5 (9.3%)	1 (4.5%)	1 (20.0%)	1 (100.0%)	0 (0.0%)	2 (25.0%)		
**Distant metastases**							0.015	0.008
Yes	24 (44.4%)	12 (54.5%)	3 (60.0%)	0 (0.0%)	3 (16.7%)	6 (75.0%)		
No	30 (55.6%)	10 (45.5%)	2 (40.0%)	1 (100.0%)	15 (83.3%)	2 (25.0%)		
Follow up	95.9 (78.6–112.7)	95.9 (75.5–110.2)	104.6 (89.6–105.4)	84.4 (84.4–84.4)	101.7 (80.8–119.8)	94.4 (87.1–100.7)	0.86	0.50
[months]								

**Table 2:** Number of genes significantly deregulated depending on the molecular differentiating factor; M- the presence of *BRAF* or *TERT*p or *RAS* somatic mutation; NM- no somatic mutations within studied genes.

Type of Comparison	Number of Differentiating Genes	
	*p* < 0.001	FDR < 0.05
M (32 PTCs) vs. NM (22 PTCs)	1404	2543
BRAF(+)TERTp(+) (8 PTCs) vs. BRAF(+)TERTp(−) (18 PTCs)	126	4
BRAF(+)TERTp(−) (18 PTCs) vs. RAS(+) (5 PTCs)	523	709
RAS(+) (5 PTCs) vs. NM (22 PTCs)	97	3

**Table 3:** Genes differentiating BRAF(+)TERTp(+) PTCs and BRAF(+)TERTp(−) PTCs.

Gene Symbol	Entrez ID	Gene Name	BRAF(+)TERTp(+) Mean	BRAF(+)TERTp(−) Mean	Fold Change	*p*-Value	Adjusted *p*-Value
*CRABP2*	1382	cellular retinoic acid binding protein 2	7.92	6.32	3.03	5.7 × 10^−7^	0.012
*ECM1*	1893	extracellular matrix protein 1	8.07	6.77	2.47	5.9 × 10^−6^	0.042
*KRT17*	3872	keratin 17	5.07	4.29	1.72	7.9 × 10^−6^	0.042
*MTMR3*	8897	myotubularin related protein 3	6.64	7.04	0.76	8.1 × 10^−6^	0.042

**Table 4:** Validation of genes differentiating BRAF(+)TERTp(+) and BRAF(+)TERTp(−) PTCs performed on an independent set of PTC made available by the TCGA study.

Gene Information		Our Study						TCGA Study		Compliance Between Our Study and TCGA Data
Gene Symbol	Entrez ID	BRAF(+)TERTp(+) Mean Expression	BRAF(+)TERTp(−) Mean Expression	*p*-Value	Fdr-Adjusted *p*-Value	Fold Change	*p*-Value	FDR-Adjusted *p*-Value	Fold Change	
*CRABP2*	1382	7.92	6.32	5.7 × 10^−7^	0.012	3.03	5.8 × 10^−8^	1.0 × 10^−5^	2.21	YES
*ECM1*	1893	8.07	6.77	5.9 × 10^−6^	0.042	2.47	0.33	0.69	1.27	NO
*KRT17*	3872	5.07	4.29	7.9 × 10^−6^	0.042	1.72	0.10	0.41	0.53	NO
*MTMR3*	8897	6.64	7.04	8.1 × 10^−6^	0.042	0.76	0.11	0.43	0.92	NO

**Table 5:** Gene groups (pathways and gene ontology terms) significantly differentiating BRAF(+)TERTp(+) and BRAF(+)TERTp(−) PTCs obtained by the Gene Set Enrichment Analysis (FDR < 0.05). Ten gene groups with the highest NES were selected from each analysis.

**KEGG Pathways**					
**ID**	**Description**	**Set Size**	**NES**	***p*-Value**	**Adjusted *p*-Value**
hsa00562	Inositol phosphate metabolism	74	−2.57	0.00366	0.04234
hsa04070	Phosphatidylinositol signaling system	99	−2.38	0.00408	0.04234
hsa04120	Ubiquitin mediated proteolysis	134	−2.16	0.00500	0.04234
hsa04080	Neuroactive ligand-receptor interaction	333	2.11	0.00112	0.04234
hsa04950	Maturity onset diabetes of the young	26	2.03	0.00156	0.04234
hsa04740	Olfactory transduction	411	1.99	0.00110	0.04234
hsa04141	Protein processing in endoplasmic reticulum	162	−1.96	0.00510	0.04234
hsa05217	Basal cell carcinoma	63	1.92	0.00141	0.04234
hsa04666	Fc gamma R-mediated phagocytosis	87	−1.83	0.00389	0.04234
hsa04744	Phototransduction	28	1.83	0.00470	0.04234
**Gene Ontology Biological Process**					
**ID**	**Description**	**Set size**	**NES**	***p*-Value**	**Adjusted *p*-Value**
GO:0046856	phosphatidylinositol dephosphorylation	22	−2.63	0.00029	0.01786
GO:0031424	Keratinization	208	2.57	0.00012	0.01786
GO:0032508	DNA duplex unwinding	73	−2.56	0.00038	0.01786
GO:0006261	DNA-dependent DNA replication	127	−2.54	0.00047	0.01786
GO:0046839	phospholipid dephosphorylation	27	−2.53	0.00030	0.01786
GO:0045005	DNA-dependent DNA replication maintenance of fidelity	32	−2.49	0.00031	0.01786
GO:0032878	regulation of establishment or maintenance of cell polarity	23	−2.49	0.00029	0.01786
GO:0050853	B cell receptor signaling pathway	50	−2.47	0.00034	0.01786
GO:0006895	Golgi to endosome transport	14	−2.47	0.00027	0.01786
GO:2000114	regulation of establishment of cell polarity	21	−2.47	0.00029	0.01786
**Gene Ontology Cellular Components**					
**ID**	**Description**	**Set size**	**NES**	***p*-Value**	**Adjusted *p*-Value**
GO:0005882	intermediate filament	180	2.41	0.00012	0.00681
GO:0045095	keratin filament	84	2.36	0.00013	0.00681
GO:1990234	transferase complex	586	−2.25	0.00225	0.01802
GO:0042555	MCM complex	11	−2.16	0.00026	0.00894
GO:0010494	cytoplasmic stress granule	54	−2.16	0.00035	0.00894
GO:0005681	spliceosomal complex	155	−2.15	0.00055	0.00894
GO:0030990	intraciliary transport particle	22	−2.15	0.00029	0.00894
GO:0045111	intermediate filament cytoskeleton	218	2.14	0.00012	0.00681
GO:0016607	nuclear speck	377	−2.14	0.00116	0.01224
GO:0000123	histone acetyltransferase complex	72	−2.13	0.00038	0.00894
**Gene Ontology Molecular Function**					
**ID**	**Description**	**Set size**	**NES**	***p*-Value**	**Adjusted *p*-Value**
GO:0004386	helicase activity	125	−2.86	0.00049	0.00730
GO:0052866	phosphatidylinositol phosphate phosphatase activity	25	−2.73	0.00029	0.00730
GO:0061733	peptide-lysine-N-acetyltransferase activity	50	−2.65	0.00035	0.00730
GO:0034212	peptide N-acetyltransferase activity	55	−2.62	0.00036	0.00730
GO:0004402	histone acetyltransferase activity	48	−2.61	0.00034	0.00730
GO:0034593	phosphatidylinositol bisphosphate phosphatase activity	20	−2.60	0.00029	0.00730
GO:0008094	DNA-dependent ATPase activity	70	−2.52	0.00038	0.00730
GO:0003678	DNA helicase activity	41	−2.52	0.00033	0.00730
GO:0005096	GTPase activator activity	194	−2.50	0.00065	0.00778
GO:0005085	guanyl-nucleotide exchange factor activity	193	−2.47	0.00064	0.00778

**Table 6:** Significantly enriched gene groups (FDR < 0.05) related to telomeres.

**Gene Ontology Biological Process**					
**ID**	**Description**	**Set Size**	**NES**	***p*-Value**	**Adjusted *p*-Value**
GO:0032204	regulation of telomere maintenance	75	−1.81	0.00077	0.01884
GO:0032205	negative regulation of telomere maintenance	32	−1.79	0.00312	0.03647
GO:0000723	telomere maintenance	147	−1.72	0.00052	0.01786
GO:0032200	telomere organization	159	−1.57	0.00162	0.02471
**Gene Ontology Cellular Components**					
**ID**	**Description**	**Set Size**	**NES**	***p*-Value**	**Adjusted *p*-Value**
GO:0000781	chromosome, telomeric region	149	−1.67	0.00053	0.00894
GO:0000784	nuclear chromosome, telomeric region	115	−1.55	0.00559	0.03593

## Supplementary Materials

The following are available online at https://www.mdpi.com/2072-6694/12/6/1597/s1, Table S1: The list of *KRAS*, *NRAS*, *HRAS* and *BRAF* mutations detectable by the Thyroid Cancer Mutation Analysis Kit (THDNA-RT64; EntroGen), Table S2: Lists of genes significantly deregulated depending on the molecular differentiating factor; M- presence of *BRAF* or *TERT*p or *RAS* somatic mutation; NM- lack of somatic mutations within studied genes.
